# The Effects of a Virtual Reality–Based Training Program for Adolescents With Disruptive Behavior Problems on Cognitive Distortions and Treatment Motivation: Protocol for a Multiple Baseline Single-Case Experimental Design

**DOI:** 10.2196/33555

**Published:** 2022-05-20

**Authors:** Renée E Klein Schaarsberg, Arne Popma, Ramón J L Lindauer, Levi van Dam

**Affiliations:** 1 Child and Adolescent Psychiatry & Psychosocial Care Amsterdam UMC location Vrije Universiteit Amsterdam Amsterdam Netherlands; 2 Dutch Innovation Network for Societal Youth Challenges Garage2020 Amsterdam Netherlands; 3 Mental Health Amsterdam Public Health Amsterdam Netherlands; 4 Academic Center for Child and Adolescent Psychiatry Levvel Amsterdam Netherlands; 5 Child and Adolescent Psychiatry Amsterdam UMC location University of Amsterdam Amsterdam Netherlands; 6 Department of Child Development and Education University of Amsterdam Amsterdam Netherlands

**Keywords:** treatment motivation, cognitive distortions, reflective functioning, disruptive behavior problems, adolescence, virtual reality, single-case experimental design

## Abstract

**Background:**

Serious disruptive behavior among adolescents is a prevalent and often persistent problem. This highlights the importance of adequate and effective treatment to help adolescents with disruptive behavior problems react less hostile and aggressive. In order to create a treatment environment in which behavioral change can be enhanced, treatment motivation plays an essential role. Regarding treatment itself, a focus on challenging self-serving cognitive distortions in order to achieve behavioral change is important. Street Temptations (ST) is a new training program that was developed to address both treatment motivation and cognitive distortions in adolescents with disruptive behavior problems. One of the innovative aspects of ST is the use of virtual reality (VR) techniques to provide adolescents during treatment with visually presented daily social scenarios to activate emotional engagement and dysfunctional cognitions. By using the VR scenarios as an integral starting point of ST’s sessions and transferring the power of the VR experience into playful and dynamic exercises to practice social perspective–taking, adolescents are encouraged to reflect on both their own behavior and that of others. This focus on reflection is grounded in ST’s main treatment mechanism to influence treatment motivation and cognitive distortions, namely, mentalizing (ie, reflective functioning).

**Objective:**

The aim of this study is to describe the research protocol to evaluate the effects of ST on treatment motivation and cognitive distortions. We take a closer look at the use of ST and the methodology used, namely, the repeated single-case experimental design (SCED).

**Methods:**

The effects of ST are studied through a multiple baseline SCED, using both quantitative and qualitative data. In total, 18 adolescents from secure residential youth care facilities and secondary special education schools are randomly assigned to 1 of the 3 different baseline conditions. Throughout the baseline phase (1, 2, or 3 weeks), intervention phase (4 weeks), and follow-up phase (1, 2, or 3 weeks), daily measurements on treatment motivation and cognitive distortions are conducted. Secondary study parameters are assessed before baseline, after intervention, and after follow-up. Qualitative data are collected after intervention, as well as at 3 months and 6 months after the intervention.

**Results:**

Data collection for this study started in November 2021 and is planned to be completed by August 2023. The results will be published in peer-reviewed journals and presented at national and international conferences.

**Conclusions:**

ST aims to improve the disruptive behavior problems of adolescents. This study will be the first to gain insights into the effectiveness of ST. The strengths of this study include its thorough and individually focused design (SCED), the focus on a residential as well as a secondary special education setting, and the ecological validity. The implications for practice are discussed.

**Trial Registration:**

Central Committee on Research Involving Human Subjects NL75545.029.20. Netherlands Trial Register NL9639; https://www.trialregister.nl/trial/9639

**International Registered Report Identifier (IRRID):**

PRR1-10.2196/33555

## Introduction

### Background

Disruptive behavior of young children is among the most frequent reasons for referral to child and adolescent mental health care services worldwide [1]. During adolescence, disruptive behavior continues to be a widely acknowledged problem [2-5]. Disruptive behavior disorders include conduct disorder and oppositional defiant disorder [6]. Behavior that fits the classification of disruptive behavior disorders can be characterized as disobedient, stubborn, irritable, or even hostile and aggressive, and often manifests itself in patterns of uncooperative and defiant behavior [6]. Adolescents themselves are affected by displaying such behavior as well as their surroundings and society as a whole [7-9]. Consistent occurrence of untreated disruptive behavior problems has a wide variety of persistent negative outcomes such as school dropouts, substance use, developing antisocial personality disorders, and both nonviolent and violent delinquency and criminality [7,10-13]. This highlights the importance of adequate and effective treatment for adolescents with disruptive behavior problems in order to help them develop and increase the necessary skills to react in a less hostile and aggressive way.

Well-established, evidence-based treatments for adolescents with disruptive behavior problems, such as the Multisystemic Therapy and Treatment Foster Care Oregon, primarily focus on a small portion of adolescents with disruptive behavior problems, that is, adolescents with judicial involvement within the context of forensic youth care [14]. Compared to more typical disruptive adolescents, this subpopulation of adolescents with judicial involvement tends to show a significantly higher severity of problems and needs [14]. Although well-established treatments aimed at these more severe problems and needs are available, as mentioned, the effects of such programs appear to be smaller than the effects of more preventive programs targeting adolescents at the onset of judicial involvement [15]. In other words, intervening at an earlier stage seems to be more effective than curing at a later stage. It is safe to say that adequate treatment options for adolescents with less severe disruptive behavior problems are also needed in order to prevent the escalation of these problems.

Treatment motivation is considered to be one of the preconditions for treatment to be effective [16,17]. Interventions that theoretically have the right focus may still have difficulty accomplishing progress when adolescents’ resistance to treatment is not addressed as well [18]. Motivation and involvement of all key players were also found to be one of the common treatment mechanisms in the evidence-based systemic treatments mentioned above [19]. However, lack of treatment motivation is relatively common among adolescents with disruptive behavior problems [20-23]. Consequently, a focus on intrinsic motivation is an important factor in providing the opportunities for enduring behavior changes in adolescents [24]. By implementing programs or modules that increase adolescents’ motivation, the chances of successful treatment can be increased [25]. More specifically, a study by van der Stouwe et al [26] showed treatment motivation to be predictive of self-serving cognitive distortions in a sample of Dutch juvenile delinquents. Juveniles showed better results for these outcomes when their motivation was higher, regardless of the treatment condition.

The self-serving cognitive distortions mentioned above are associated with disruptive behavior problems [27-31]. Cognitive distortions are defined as “inaccurate or biased ways of attending to or conferring meaning upon experiences” [32], because of which problematic emotional responses and behavior can arise [31]. The primary self-serving cognitive distortions, that is, self-centered distortions, indicate that someone considers their own views, expectations, needs, rights, immediate feelings, and desires to be of such importance that someone else’s legitimate views (or even one’s own long-term best interest) are scarcely considered or disregarded altogether [27]. These primary distortions increase the chance of engaging in disruptive behavior [33]. They are typically accompanied by 3 types of secondary cognitive distortions that function as protective rationalizations against certain types of psychological stress [18,33]. These are categorized as blaming others, assuming the worst, and minimizing or mislabeling [32]. A specific biased way of attributing meaning that has been given prominent attention in research is hostile attribution bias (HAB) [34]. HAB can be seen as an example of the category assuming the worst [31,32,35]. Research shows that self-serving cognitive distortions and HAB more specifically can improve when targeted during treatment [26,33,36,37].

According to Gibbs et al [18], challenging and encouraging adolescents to put themselves in others’ positions directly challenges adolescents’ self-serving cognitive distortions as well. Providing social perspective–taking opportunities should thus play a fundamental role when treating adolescents with disruptive behavior problems [18]. Research by Verhoef et al [34] implies that these social perspective–taking opportunities should be primarily targeted at emotionally engaging situations. Their meta-analysis showed that the relation between HAB and aggressive behavior was stronger in social interactions that evoked sufficient emotional engagement. Inhibiting deliberate reflective processing by derailing cognitive processes, the strong emotions may elicit the automatic and emotional processes that activate HAB [34]. When activated, the needed content to work with during treatment sessions emerges.

Taken together, adequate treatment options for adolescents with disruptive behavior problems are needed to prevent escalation of their problems. Treatment motivation is an important requisite to increase the chances of successful behavior change. In terms of content, emotionally engaging social perspective–taking opportunities can challenge self-serving cognitive distortions and in that way induce behavioral change.

### Street Temptations

Street Temptations (ST) is a new and innovative training program that was developed by Garage2020 in cocreation with Levvel, a secure residential facility and youth care provider in Amsterdam, The Netherlands, to influence treatment motivation as well as cognitive distortions of adolescents with disruptive behavior problems. In order to achieve this effect, ST’s exercises focus entirely on social perspective–taking opportunities. ST specifically aims to work with scenarios that are emotionally engaging, as this is the type of situation that should be focused on in treatment [34]. To create these scenarios, ST uses the potential of virtual reality (VR).

The term VR indicates a replacement of the physical environment by a 3D computer or an artificially generated interactive environment [38]. When done right, VR has the power to achieve full immersion and presence [39,40]. Immersion refers to the degree to which a user is aware of the real world while in the VR environment. Presence refers to “the psychological state in which a participant accepts, interacts, and is physically, socially, and emotionally engaged in the virtual world” [41]. Put otherwise, presence causes the subjective sense of “being there” [42,43]. Together, immersion and presence will let the human brain treat a VR experience as psychologically real, letting users react toward the VR experience as if it were real [39]. In this way, VR ensures there is less of a demand on the cognitive abilities needed to make a realistic representation of a hypothetical situation [44]. This makes VR ideally suited to meet the needs of the adolescents with disruptive behavior problems aimed at, considering that mild-to-borderline intellectual disabilities are not uncommon within this target population [45,46]. Visual support is highly recommended when treating children and adolescents with mild-to-borderline intellectual disabilities [47]. Consequently, this innovative feature can provide the necessary emotional engaging scenarios for social perspective–taking challenges. Additionally, with VR, it is possible to create realistic and recognizable scenarios that in the real world would be impossible or unethical to create [39]. The power of the VR scenarios is extended in playful and dynamic exercises. By providing therapists with practical tools, they are enabled to encourage adolescents to reflect on both their own behavior as on that of others.

The focus on reflection is grounded in the assumably main therapeutic mechanism of ST, that is, mentalizing. The concept of mentalizing, operationalized as reflective functioning, refers to “the mental process by which an individual implicitly and explicitly interprets the actions of himself or herself and others as meaningful on the basis of intentional mental states such as personal desires, needs, feelings, beliefs, and reasons” [48]. Through this mental process, people can make sense of their social world, making mentalization a core aspect of human social functioning [49]. Research shows that many adolescents with disruptive behavior problems have difficulty mentalizing [50]. Mentalizing problems cause difficulties in predicting and anticipating the behavior and motives of others [51-53]. Problems regarding self-awareness and self-regulation are likely to occur as well [49]. Consequently, the risk of misunderstanding social cues and impulsive actions within the context of interpersonal communication increases [50]. An empirical evaluation by Bo et al [54] shows that the mentalizing abilities of adolescents with diagnosed borderline personality disorder can significantly improve over the course of mentalization-based treatment.

Since ST is a newly developed program, so far, only test runs regarding the feasibility and potential of the program have been conducted [55]. Owing to the importance of adequate treatment programs for adolescents with disruptive behavior problems as well as creating the conditions under which the likelihood of successful treatment increases, more extensive research into the value and effectiveness of ST is needed. The aim of this study is to describe the repeated single-case experimental design (SCED) that is used to provide a first and thorough exploration into ST’s effectiveness.

## Methods

### Design

This study applies a randomized, nonconcurrent, multiple baseline SCED across single participants [56,57]. As compared to group-comparison designs where experimental units refer to groups of participants assigned to different conditions, the experimental units in SCEDs are formed by repeated measurements within an individual [58]. In a multiple baseline SCED, repeated measurements are conducted both in the absence and in the presence of a certain treatment. This allows participants to serve as their own controls [59]. Well-designed and well-executed SCEDs are able to determine whether a causal relationship exists between an intervention (ie, an independent variable) and change in an outcome measure (ie, a dependent variable) [60]. SCEDs can be particularly useful in the early developmental phase of research, whereby unforeseen adjustments can be implemented immediately [59,61]. Additionally, SCEDs allow tailoring the intervention to the unique needs of participants. SCEDs also lend themselves very well for research in clinical settings, with small and heterogeneous populations that are difficult to capture in more standard group designs such as randomized controlled trials. The population ST focuses on can be characterized as such a population. Moreover, the intense and comprehensive studying of a small number of participants allows better knowledge of the studied individuals, insight into possible mediation effects, and the detection of intervention effects within the variability of participants’ performances [62].

**Figure 1:**
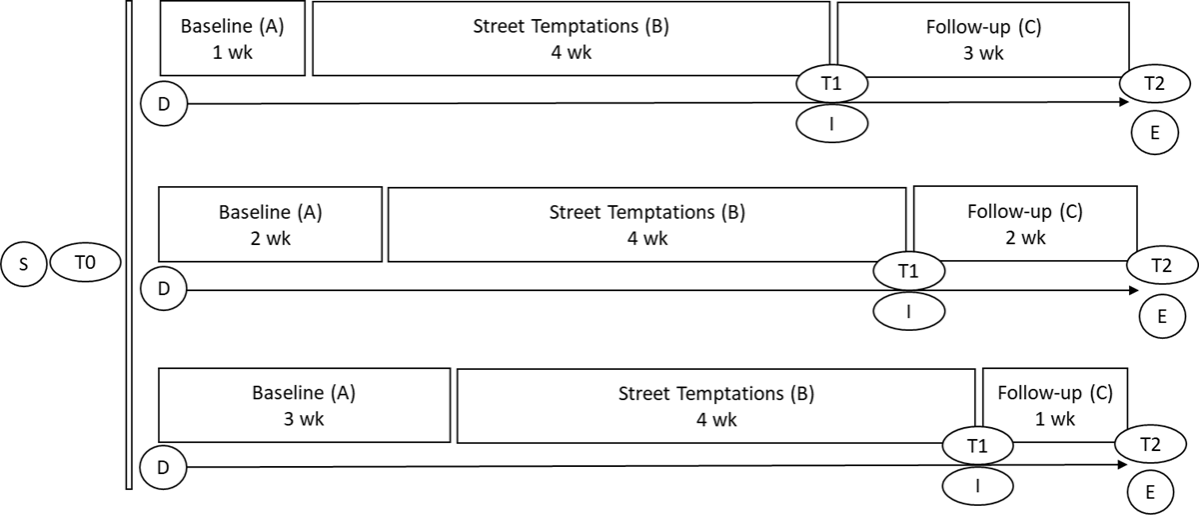
Overview of the study design with 3 different conditions. The daily repeated measure starts directly after T0, on the same day. Moreover, 3 and 6 months after T2, adolescents are approached again to participate in follow-up interviews. D: daily repeated measure; E: end of daily measure study period; I: first interview with adolescents and trainers; S: start of the study, application, informed consent, and eligibility check; T0: pretreatment assessment and randomization; T1: posttreatment assessment; T2: short-term follow-up assessment; wk: week.

Participants are randomized to a 1-, 2-, or 3-week baseline phase. Randomization to varying baseline periods enables us to determine whether change in measurements is exclusively related to the application of the intervention. The random assignment is similar to the way in which a random assignment is used in between-participants designs [58]. The length of the phases has been chosen to keep them as short as possible in order to prevent dropout of the already difficult-to-reach target population. To assess primary outcomes, participants complete repeated measurements during a baseline phase (phase A), an intervention phase (phase B), and a short-term follow-up phase (phase C). These measurements are administered electronically once a day using a smartphone app. Phase A acts as a control and is therefore compared to phases B and C. To assess secondary outcomes, pre-(T0), post-(T1), and short-term follow-up measurements (T2), as well as qualitative data collection are used within the SCEDs. In total, the research period from the start of the baseline until the end of the short-term follow-up takes up approximately 8 weeks per participant. In addition to the short-term follow-up, qualitative data are also collected at 3 months and 6 months after T2. An overview of the study design is shown in Figure 1.

### Participants

Participants are recruited among adolescents from secure residential youth care facilities and secondary special education schools in the Netherlands. Both populations are characterized by serious externalizing problems. These problems are often accompanied by internalizing problems, sometimes in combination with psychiatric and addiction problems. Adolescents who meet the following criteria are eligible for inclusion: (1) aged between 12 and 18 years, (2) antisocial or externalizing behavioral problems, (3) deficits regarding cognitive distortions or treatment motivation, (4) presence or risk of delinquent behavior, (5) assigned to ST after multidisciplinary consultation, (6) expected stay of at least 2 months, and (7) basic understanding of mobile apps. A potential participant who meets any of the following criteria is excluded from participation: (1) severe physical impairment such as deafness and blindness, (2) severe psychiatric problems such as psychosis or high risk of suicide requiring immediate intervention, (3) trauma from serious violence, (4) epilepsy or serious problems regarding motion sickness, and (5) insufficient understanding of the spoken and written Dutch language.

### Sample Size

**Figure 2:**
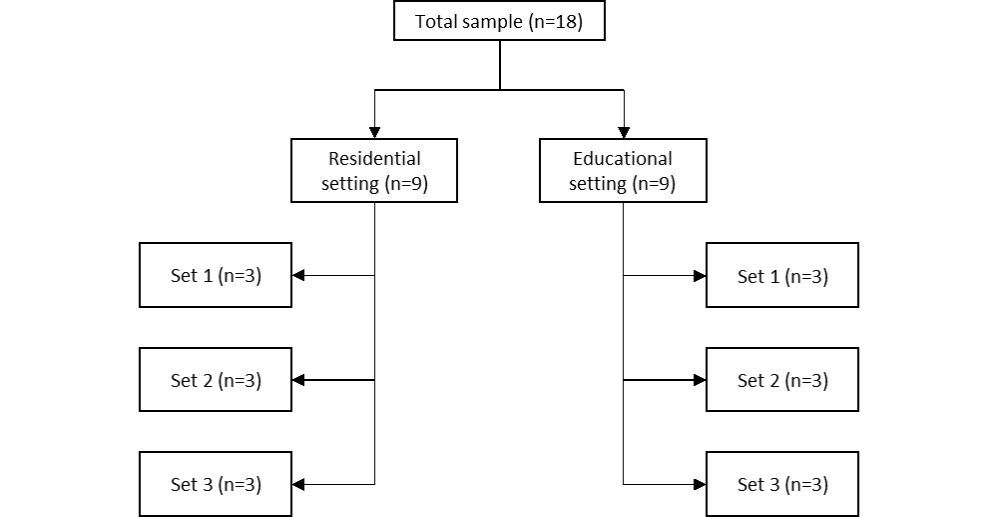
Overview of the sample distribution.

According to SCED research standards, SCEDs need at least 3 attempts to demonstrate an intervention effect. Each of these attempts needs to be at a different time point, requiring a multiple baseline SCED to have at least 3 baseline conditions. Additionally, each phase must include a minimum of 3, preferably 5, data points to qualify as an attempt to demonstrate an effect [59,63,64]. Regarding treatment attrition, a meta-analysis on inpatient juvenile offender treatment showed attrition rates of up to about 60% [17]. A study on residential treatment for adolescents with serious disruptive behavior problems reported that 51% had left the treatment center prematurely [65]. In order to adhere to the stated minimum of 3 participants by SCED research standards, while compensating for a potential 60% attrition rate, a total of 18 participants is strived to include in the study. The 18 participants are equally divided over the 2 research settings and the 3 baseline conditions. In combination with the daily repeated measurements, this sample results in 6 initial attempts to demonstrate an intervention effect of ST. An overview of the sample distribution is displayed in Figure 2. Each set consists of the 3 baseline conditions shown in Figure 1.

### Procedures

Within the residential facilities, all referred adolescents are screened by the clinicians and ST trainers. Regarding the participating schools, adolescents from selected classes are screened during the first few weeks of the new academic year. This screening process is chosen to minimize the risk of nonresponse. Screening is only done by the professionals from the facilities and schools. All professionals involved are extensively briefed on the population the study focuses on. When an adolescent is thought to be eligible to participate in the study, the professional informs the adolescent about the study. The adolescent is shown a short video, in which the researcher briefly introduces herself and the project. Written information is provided as well. When the adolescent is interested to participate and gives oral permission to be approached, the researcher plans an informed consent appointment. Written informed consent is signed when the adolescent agrees to participate in the study. In the case of a minor, parents or a legal guardian signs a written informed consent as well. Adolescents that do not agree to participate in the study do not start with ST and receive treatment as usual.

After the informed consent procedure, the researchers decide whether a participant is eligible to take part based on the inclusion and exclusion criteria. When necessary, a clinician will be consulted to make an informed decision. After official enrollment, premeasurements are conducted, and randomization takes place. Because trainers, clinicians, and adolescents will notice in which condition adolescents are participating, allocation is not blinded. Appointments for the ST-intake session and following sessions are made according to the randomization. Directly after the premeasurements have been conducted, the daily measurements are set up by installing the data collection app (m-Path). Everything about the app and data collection is explained as well as tested with the adolescent. From then on, the app automatically sends out notifications for the daily assessments within set time frames. This continues until the last day of the follow-up phase, according to the randomization. During the baseline and intervention phase, the researcher, ST trainers, and participants are in touch on a regular basis. Together, they check how things are going and whether there are any particularities regarding ST, the daily measurements, or in general. The intensity (eg, frequency, duration) of these contact moments will be kept the same across participants as much as possible. If necessary, for example, to encourage participants to fill in their daily measurements, the researcher will be in touch more often. Contact moments will be registered per participant in order to take possible variations regarding contact moments into account when analyzing treatment effects.

ST trainers inform the researcher when the last ST session takes place. After the last session has taken place, 2 appointments are scheduled with the participating adolescent: 1 for the posttreatment assessment and 1 for the interview. This is done separately in order to reduce the burden on the adolescent. Additionally, 1 appointment is scheduled with the ST trainer for the interview with the trainer. After the last ST session, participants enter the follow-up phase. The researcher schedules the last appointment at the end of this phase to conduct the follow-up measurements and to close the study period together with the ST trainer and participant. At 3 months and 6 months after the end of the study period, the adolescents are approached again to participate in the 2 additional follow-up interviews. When a participant decides to leave the study prematurely, it is possible to finish the remaining ST sessions. The decision to do so or not will always be made in consultation with the trainer and clinician. Owing to the inevitable heterogeneity with regard to both the problems of the adolescents as well as the moment at which they participate in ST within their treatment process, no restrictions are imposed with regard to cointervention.

### ST Sessions

ST consists of seven 45-60-minute sessions. During the sessions, input and direction from the adolescents form the main lead regarding the exercises to offer adolescents a creative and alternative way to develop certain skills and to freely share their personal story. In this way, ST aims to add to more traditional modes that are not always tailored to the needs of adolescents [66]. This adheres to adolescents’ need for autonomy and control regarding their treatment [67]. Adolescents underscore their own voices and contributions as essential for successful therapy in order for therapists to really get to know their personal stories [68].

By incorporating mentalizing as the main therapeutic mechanism, ST aims to influence both treatment motivation and cognitive distortions. In order to develop the needed motivation to engage in treatment for behavioral change, it is in the first place necessary to acknowledge problematic behavior and to seek help for this behavior [69]. Additionally, someone must have the desire to behave more socially adequate and to formulate the kind of person someone would want to be in the future. Clustering these factors, te Velde et al [70] describe self-reflection as an important concept regarding treatment motivation. Since self-reflection is part of the definition of mentalizing, it is plausible that mentalizing can function as a therapeutic mechanism regarding treatment motivation. Besides, social perspective–taking is an important component of mentalizing [71]. By centering the exercises around that component, the incorrect or biased ways of attending or conferring meaning upon experiences (ie, the cognitive distortions) can be challenged [18]. Given its definition, the act of mentalization reflects the way in which an individual can give meaning to social experiences, namely, on the basis of inner mental experiences. By giving that way of meaning making a central role within the exercises and thereby helping adolescents improve the way they can make sense of their social world, mentalizing can function as a therapeutic mechanism regarding cognitive distortions as well.

The sessions are divided over 2 modules, A and B, which are executed in a fixed order. Before starting with the first module (ie, A), there is an intake session during which the adolescent chooses a personal learning goal. This goal focuses on mentalizing abilities, for example, “I want to learn that how I see a situation doesn’t have to be the same as how somebody else sees the same situation” or “I want to learn to listen to what somebody else thinks, feels, or would want to do in a situation so that I can better understand that person.” Each consecutive session ends with discussing the personal learning objective.

### Modules of the Sessions

#### Module A

**Figure 3:**
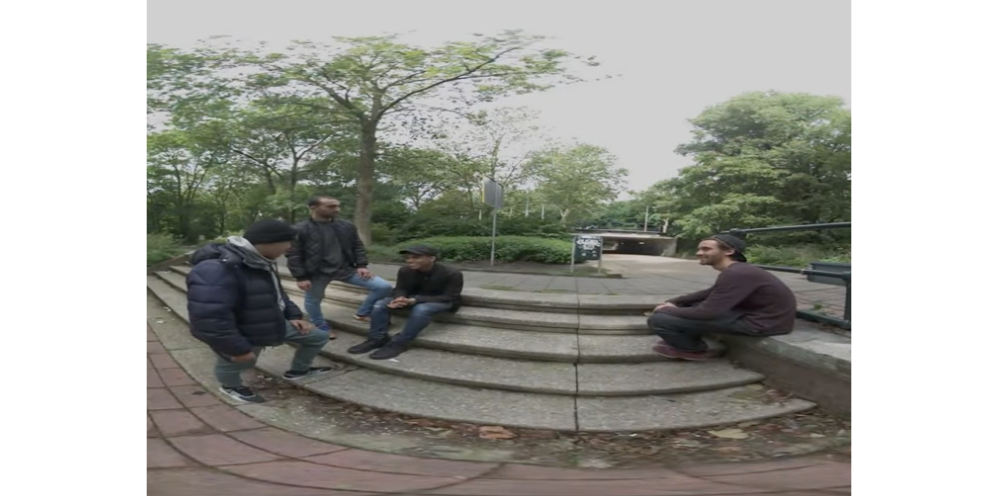
Screen capture of the virtual reality video.

**Figure 4:**
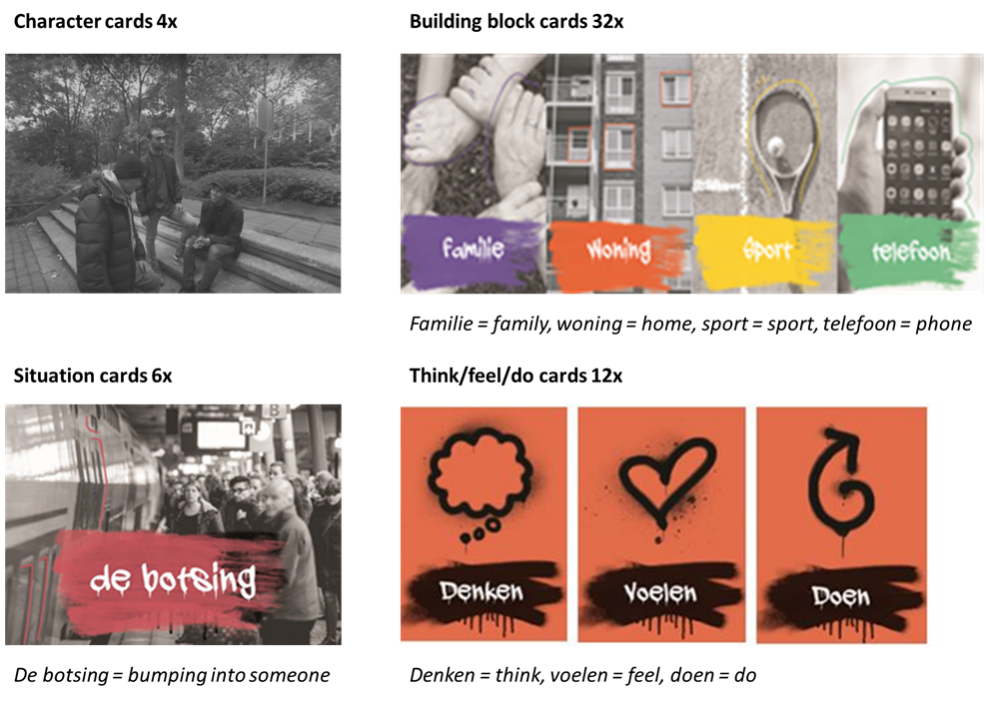
Examples of the cards used in the sessions.

Module A revolves around 3 main characters. Each session starts with watching a 360° VR video. This video (see Figure 3) is used to present the scenario and characters on which the exercises are based. The video shows a small group of guys in a park. Youngster 1 forces youngster 2 to beat up a passerby, youngster 3, and youngster 2 obeys. In between, there are fragments shown in which youngster 2 is interviewed about why he knocked down youngster 3. The video ends with a compilation of videos from the internet of real fights between adolescents. During watching, the video is streamed from the VR glasses to an extra screen, allowing the trainer to see what the adolescent is watching simultaneously. After watching the video, the adolescent chooses one of the 3 characters to focus on that session. Together with the trainer, the adolescent creates a backstory for the character based on various building block cards (see Figure 4), for example, for family, living situation, and sports. When the character has been given a personal story, the adolescent takes on the character’s perspective. Based on that perspective, linked to the created personal story, the trainer challenges the adolescent to reflect on the scenario from the video. After that, the adolescent switches back to their own perspective to discuss the differences and similarities between the 2 perspectives and why those might be present. Apart from watching the VR video, the exercises are performed outside of the VR environment. Figure 4 shows examples of the different cards that are used.

#### Module B

In module B, the exercises revolve mainly around a personal experience chosen by the adolescent. Adolescents visualize the scenario of this situation for the trainer by using Street View VR, which means that the adolescent will use the VR glasses to virtually go to the place of their personal experience. While virtually being present in that place, the trainer watches along with the stream of the VR glasses and the adolescent explains what exactly happened in that place. Thereafter, the same perspective-switching exercises as described above are executed but with different perspectives. One session is about the perspective from an unknown passerby, and the other session is about the perspective from someone in the social network of the adolescent. In an additional exercise, a fictional character is created. This character is put in different situations based on the situation cards, and the adolescent has to make and substantiate a choice in that situation based on the character. As in module A, in module B, all exercises take place outside of the VR environment as well. The use of VR serves to present a scenario on which the exercises will be based. During the last session, the trainer and adolescent reflect on the progress that has been made regarding the personal learning goal. They also evaluate the program all together and what the adolescent has learned in addition to the set learning objective. It is possible to involve, for example, the mentor in this final session and have the adolescent explain what has been done and learned.

### Training and Supervision

ST trainers receive a 2-day training course, provided by the first author and a highly experienced psychotherapist who is also a registered teacher and supervisor. The training focuses on the theoretical background of ST, working with VR, the ST protocol, and practicing the learned skills by participating in and reflecting on role plays with experience experts. In addition to the training, ST trainers are guided throughout the research period by participating in monthly supervision sessions. These sessions are also facilitated by the first author and the clinician from the ST training. Besides the supervision, trainers are encouraged to engage in peer consultation. Lastly, they are able to receive telephonic consultation by the first author or clinician on request. To gain insight into the extent to which trainers commit to the protocol, trainers are required to fill out session forms.

### Measures

#### Primary Outcome Measures

The main study parameters are assessed once a day in the format of an idiographic digital self-report questionnaire for the adolescents. The items are based on the questionnaires that are assessed at T0 and will be presented in a random order each day.

#### Treatment Motivation

Treatment motivation is measured using the Dutch Adolescent Treatment Motivation Questionnaire (ATMQ) [72]. The ATMQ consists of 11 self-report items with a 3-point Likert scale, ranging from “not true” to “true.” Reliability and validity proved to be good [72]. For the daily questionnaire, the ATMQ is included as a whole.

#### Cognitive Distortions

Cognitive distortions are assessed using the self-report How I Think questionnaire (HIT) [32]. The HIT contains 54 six-point Likert items that vary from “totally agree” to “totally disagree.” The Dutch version of the HIT showed acceptable reliability and validity [31,73]. To create the daily questionnaire, the subscale with the highest score will be selected.

#### Secondary Outcome Measures

The secondary study parameters include change in reflective functioning and social perspective–taking as well as a qualitative exploration of the overall experiences with regard to ST and VR.

#### Reflective Functioning

Reflective functioning is measured using the Reflective Functioning Questionnaire for Youths (RFQY) [74] and the Self-Reflection and Insight Scale for Youth (SRIS-Y) [75]. The RFQY is a 46-item self-report measure scored on a 6-point Likert scale ranging from “strongly disagree” to “strongly agree.” The questionnaire is adapted from the adult version, the Reflective Functioning Questionnaire [76], by rewording some items for a better developmental match. Both studies report preliminary support regarding reliability and validity. The RFQY consists of 2 scales, with a total RFQY score deriving from the sum of both scale scores. Higher scores indicate greater capacity for reflective function. The SRIS-Y is a 17-item, self-report questionnaire, answered on a 6-point Likert scale ranging from “disagree strongly” to “agree strongly.” The instrument consists of 2 subscales, Self-Reflection and Insight, resulting in 2 separate scores. The original adult version is reported as a reliable and valid measure of self-reflection and insight in adults [77]. Likewise, the SRIS-Y appears as a developmentally appropriate and psychometrically sound measure of self-reflection and insight in adolescents [75].

#### Social Perspective–Taking

Social perspective–taking is assessed using the Perspective Taking subscale of the Interpersonal Reactivity Index [78,79]. The Perspective Taking subscale consists of 7 items, answered on a 5-point Likert scale. The Dutch version of the Interpersonal Reactivity Index appears to be a psychometric adequate instrument [80].

#### ST Evaluation

The ST evaluation is done by conducting semistructured interviews with adolescents as well as trainers based on the Change Interview [81]. The purpose of the Change Interview is to obtain information about clients’ understanding about what has changed during therapy, why those changes have occurred, and what factors might have gotten in the way of change. By obtaining this information regarding ST, the interviews enable learning whether and, if so, what changes have occurred throughout ST from the adolescents’ and trainers’ perspectives. In addition, adolescents and trainers can clarify why they think those changes have occurred, referring to both therapy and extratherapy factors. Lastly, important information about hindering factors or possible negative changes regarding ST is gathered.

#### VR Evaluation

The VR evaluation is done by adding questions regarding this topic to the above-described interviews. All respondents are asked to reflect on their experience with VR in general and working with the VR material, what they believe VR did or did not add to ST, and what they think of the video used in ST. Additionally, they are asked how they think the VR component could be improved.

#### Sociodemographic Parameters

Sociodemographic information such as age, sex, education level, ethnicity, living situation, and possible experience with minor criminal activity is collected using a demographic questionnaire developed by the researchers. Information regarding diagnostic background and treatment history is collected using file information. When recent IQ data are missing, the Screener for Intelligence and Learning Disabilities [82] is administered.

**Figure 5:**
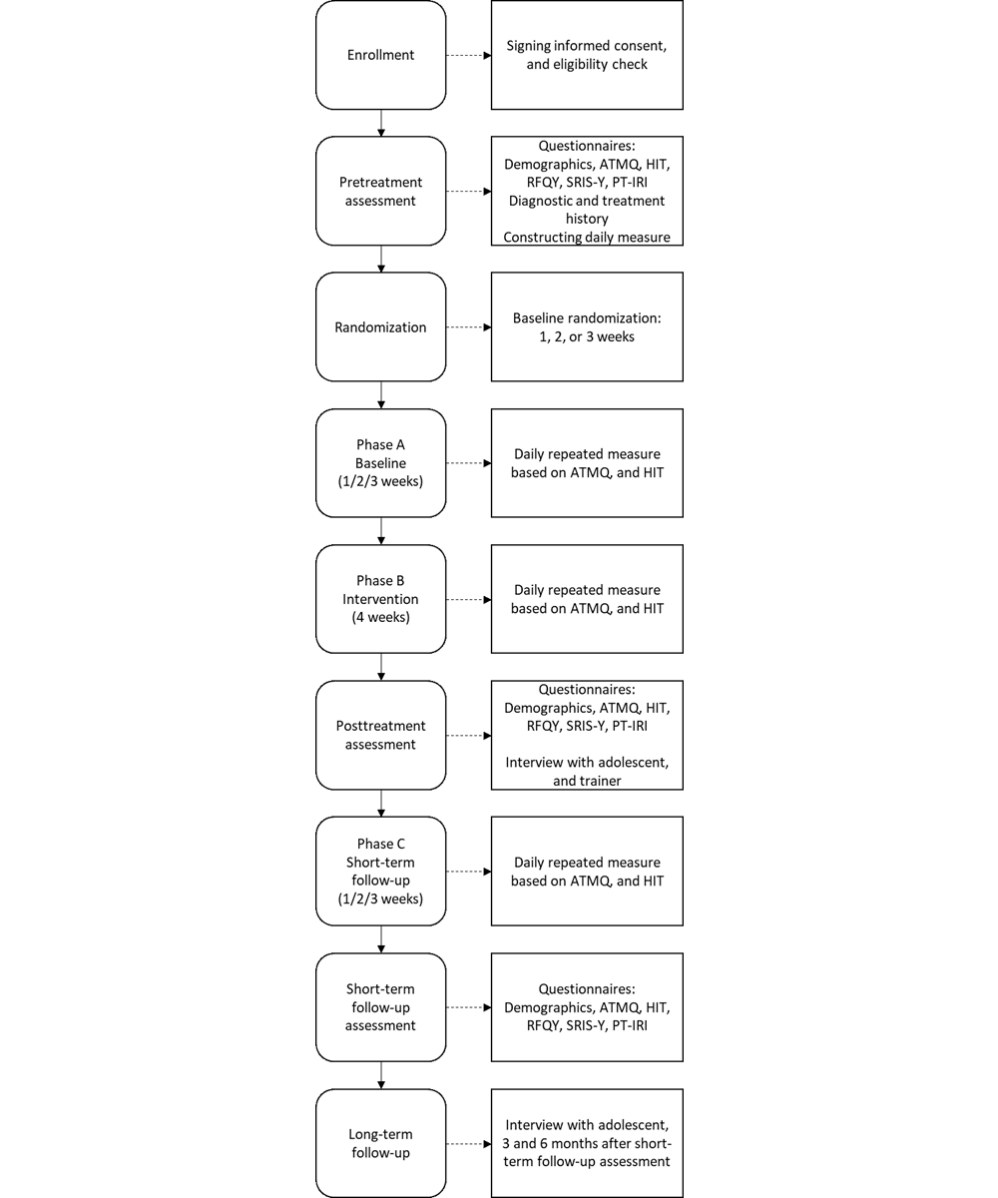
Overview of the data collection moments. ATMQ: Adolescent Treatment Motivation Questionnaire; HIT: How I Think questionnaire; IRI: Interpersonal Reactivity Index; PT: Perspective Taking; RFQY: Reflective Functioning Questionnaire for Youths; SRIS-Y: Self-Reflection and Insight Scale for Youth.

An overview of all the measurement tools and data collection moments is given in Table 1 and Figure 5.

**Table 1:** Overview of the measurement tools and informants.

Variable		Measure	Informant
**Primary outcomes**			
	Treatment motivation	ATMQ^a^: Daily questionnaire	Adolescent
	Cognitive distortions	HIT^b^: Daily questionnaire	Adolescent
**Secondary outcomes**			
	Reflective functioning	RFQY^c^, SRIS-Y^d^	Adolescent
	Social perspective-taking	PT^e^-subscale	Adolescent
	ST^f^ evaluation	Semistructured interview	Adolescent, trainer
	VR^g^ evaluation	Semistructured interview	Adolescent, trainer
**Other variables**			
	Demographics	Questions	Adolescent, clinician
	Diagnostic and treatment history	File information	Clinician
	Intelligence	File information, SCIL^h^	Clinician, adolescent

^a^ATMQ: Adolescent Treatment Motivation Questionnaire.

^b^HIT: How I Think questionnaire.

^c^RFQY: Reflective Functioning Questionnaire for Youths.

^d^SRIS-Y: Self-Reflection and Insight Scale for Youth.

^e^PT: Perspective Taking.

^f^ST: Street Temptations.

^g^VR: virtual reality.

^h^SCIL: Screener for Intelligence and Learning Disabilities.

### Analysis

The primary outcome measures are the daily self-reported questions regarding cognitive distortions and treatment motivation. The resulting data will be presented as quantitative data. Within the context of SCED, including the multiple baseline design, the primary method for data evaluation regarding these repeated measurements is visual analyses [56,57]. This means that within-participants and between-participants data will be visualized graphically in order to explore the level and rate of change between the different phases. The slopes of the variables during the intervention phase will be compared to those of the baseline and follow-up data. The overall pattern in the data will be analyzed by examining whether scores overlap across phases. In order to evaluate the reliability of potential changes, 95% confidence intervals will be calculated for each participant by using standard errors of difference. Further, a standardized mean difference effect size will be calculated for each outcome variable, using “d-statistics” for SCEDs [83]. Additionally, repeated measures analyses will be conducted. Other procedures for SCEDs will be considered if necessary. The secondary outcome measures consist of the quantitative pre-, post-, and follow-up measurements as well as the semistructured interviews conducted during posttreatment assessment and long-term follow-up. The quantitative data will be analyzed by computing a Reliable Change Index for each measure [84]. Qualitative data will be analyzed using iterative thematic analysis [85]. The interviews will be recorded using professional recording equipment in order to transcribe and analyze the data. A statistics expert of the Clinical Monitoring Center of the Amsterdam University Medical Centers (Amsterdam UMC) will be consulted regarding data analysis.

### Ethics Approval and Confidentiality of Data

Ethics approval for this study was obtained in June 2021, which was granted by the independent Medical Ethical Committee of Vrije Universiteit medical center (reference number: 2021.0114). This study will be conducted according to the principles of the World Medical Association Declaration of Helsinki [86] and in accordance with the Medical Research Involving Human Subjects Act. Handling and storage of data will be done in accordance with the General Data Protection Regulation. Collected research data within this study includes questionnaires and interviews, collected by the researchers from the Department of Child and Adolescent Psychiatry at the Amsterdam UMC. All data will be deidentified by giving every participant a unique participant ID. All data from the daily questionnaires, that is, questions and answers, are stored in a protected folder on the phone of the participant. This folder can only be accessed by the m-Path app, not by any other app. An application-layer encryption is applied to the data, meaning that the stored data itself consist of bytes without meaning. The data from these questionnaires will be transferred to the electronic case report form, captured in a custom-made Castor Electronic Data Capture database. Data from the other questionnaires will be directly collected in this database. Physical documents, for example, signed informed consent forms will be stored safely at the Department of Child and Adolescent Psychiatry at the Amsterdam UMC (location Academisch Medisch Centrum). The recordings of the interviews are stored on a protected hard disk. Research data and analyses will be stored for 15 years after finishing the research project in accordance with the Board of Directors of the Amsterdam UMC.

## Results

Participant recruitment was started in November 2021. Data collection for this study is expected to be completed by August 2023. Analysis will be conducted after data collection has been completed. The results will be published in peer-reviewed journals and presented at national and international conferences.

## Discussion

### Aims of the Study

In order to help adolescents with disruptive behavior problems develop and increase the necessary skills to react less hostile and aggressive, challenging self-serving cognitive distortions and enhancing motivation for behavioral change seem particularly important to focus on in treatment [16-18]. In this study, we have elaborated the protocol of the SCED study designed to explore ST’s effectiveness—an innovative and dynamic training program that aims to address both the mentioned focus points. Using a repeated multiple baseline SCED, we will examine the effects of participating in ST in both secure residential facilities as well as in secondary special education schools. By conducting this study, we aim to contribute to the adequate and effective treatment of disruptive behavior problems by using new and innovative treatment approaches.

### Strengths and Challenges

Our study has multiple strengths. First, by using a SCED instead of a more traditional group comparison design, a lot of individual information is collected throughout the entire treatment process while respecting each participants’ personal variability [62]. This ensures that we can gain insight into how each individual trajectory develops, allowing us to indicate for whom and under what circumstances ST is or is not of added value. Second, because the experimental units in our design are the repeated measurements within each individual adolescent instead of groups of adolescents, we are able to tailor the intervention to each adolescent’s unique needs [59,61]. This is in line with the stated importance of client-centered approaches and individually tailored treatment [68,87,88]. Third, this study does not only focus on the intensive treatment setting of secure residential care but also looks at the effects within the educational setting. Thus, this study can contribute to the essential prevention and intervention strategies in educational systems with regard to forensic youth care [89]. Overall, the use of the described design allows us to conduct thorough experimental research in the real-life circumstances of everyday clinical practice [60]. In this way, we are able to investigate the effectiveness of ST rather than the efficacy. This is an important distinction, as an efficacious intervention does not necessarily represent an effective intervention in clinical practice. Likewise, an effective intervention in clinical practice may be a less efficacious intervention in the context of scientific research [90]. Investigating ST’s effectiveness contributes to the ecological validity of our study.

In addition to the strengths, our study also poses several potential challenges. First, ST is a newly developed intervention that has not been implemented yet. We are therefore dependent on the willingness of organizations to participate and the capacity available to carry out ST in addition to the standard care that is provided. Owing to the hectic work environment of both residential care and special education settings, it may be difficult for organizations to find the time and energy to participate. To increase our chances of success, we focus on the participation of multiple organizations and locations so that our dependency is not too vulnerable. Second, although we deliberately chose a design that requires a relatively small sample size, nonresponse and dropouts are still realistic challenges. We focus on a hard-to-reach sample, and data collection demands a lot from the participating adolescents. We have tried to reduce the required effort from participants by making the daily measurement as short and easy as possible. Additionally, personal reminders will be used when assessments are not completed, and we will be in touch with participants regularly in order to keep them motivated. Third, although they are validated measures, we only use self-report questionnaires regarding the quantitative measurements. This may cause social desirability bias as well as compromise validity. However, we do use a mixed methods approach as we combine our quantitative measures with qualitative data collection. This triangulation helps us to improve the interpretation of the results and decreases the deficiency of only using self-report [91].

### Implications for Practice

ST is a new, innovative training program that specifically aims to meet the needs of adolescents by, among other things, integrating the potential of VR in the exercises. When the results are positive, ST can be further developed, implemented, and researched. In addition, when our described SCED proves to be viable for research in clinical practice, this will enhance the possibilities of clinical research. Adolescents with disruptive behavior problems usually form a hard-to-reach population, which is not easily captured in larger group designs such as randomized controlled trials. This often results in studies that are difficult to conduct, with high risks of, for example, not meeting the required sample size. This study may show alternatives for conducting good scientific research in hectic clinical environments. In this way, our study can provide both a contribution to science as well as to clinical practice.

### Conclusions

To date, no research has been conducted into the effectiveness of ST. Our study will be the first to gain insights into the value of ST in helping adolescents with disruptive behavior problems react less hostile and aggressive. Based on the results, ST can be further developed. In addition, the foundation that will be laid with this study allows us to design follow-up studies, for example, to compare the effectiveness of ST with other treatments.

## Abbreviations

ATMQ: Adolescent Treatment Motivation Questionnaire
HAB: hostile attribution bias
HIT: How I Think questionnaire
RFQY: Reflective Functioning Questionnaire for Youths
SCED: single-case experimental design
SRIS-Y: Self-Reflection and Insight Scale for Youth
ST: Street Temptations
VR: virtual reality
