# Metadata Correction: The Effects of a Virtual Reality–Based Training Program for Adolescents With Disruptive Behavior Problems on Cognitive Distortions and Treatment Motivation: Protocol for a Multiple Baseline Single-Case Experimental Design

**DOI:** 10.2196/39833

**Published:** 2022-05-27

**Authors:** Renée E Klein Schaarsberg, Arne Popma, Ramón J L Lindauer, Levi van Dam

**Affiliations:** 1 Child and Adolescent Psychiatry & Psychosocial Care Amsterdam UMC location Vrije Universiteit Amsterdam Amsterdam Netherlands; 2 Dutch Innovation Network for Societal Youth Challenges Garage2020 Amsterdam Netherlands; 3 Mental Health Amsterdam Public Health Amsterdam Netherlands; 4 Academic Center for Child and Adolescent Psychiatry Levvel Amsterdam Netherlands; 5 Child and Adolescent Psychiatry Amsterdam UMC location University of Amsterdam Amsterdam Netherlands; 6 Department of Child Development and Education University of Amsterdam Amsterdam Netherlands

In “The Effects of a Virtual Reality–Based Training Program for Adolescents With Disruptive Behavior Problems on Cognitive Distortions and Treatment Motivation: Protocol for a Multiple Baseline Single-Case Experimental Design” (JMIR Res Protoc 2022;11(5):e33555) the authors noted an error.

In the originally published article, one of the affiliations of author Ramón J L Lindauer (*Child and Adolescent Psychiatry, Amsterdam UMC Location University of Amsterdam*) was inadvertently omitted. The corrected list of authors and their affiliations now appear as follows:

Renée E Klein Schaarsberg^1,2,3^, MSc; Arne Popma^1,3,4^, MD, PhD; Ramón J L Lindauer^3,4,5^, MD, PhD; Levi van Dam^2,6^, PhD

^1^Child and Adolescent Psychiatry & Psychosocial Care, Amsterdam UMC location Vrije Universiteit Amsterdam, Amsterdam, Netherlands

^2^Dutch Innovation Network for Societal Youth Challenges, Garage2020, Amsterdam, Netherlands

^3^Mental Health, Amsterdam Public Health, Amsterdam, Netherlands

^4^Academic Center for Child and Adolescent Psychiatry, Levvel, Amsterdam, Netherlands

^5^Child and Adolescent Psychiatry, Amsterdam UMC location University of Amsterdam, Amsterdam, Netherlands

^6^Department of Child Development and Education, University of Amsterdam, Amsterdam, Netherlands

The correction will appear in the online version of the paper on the JMIR Publications website on May 27, 2022, together with the publication of this correction notice. Because this was made after submission to PubMed, PubMed Central, and other full-text repositories, the corrected article has also been resubmitted to those repositories.

